# Editorial: Theranostics as a driving force in nuclear medicine

**DOI:** 10.3389/fonc.2024.1468357

**Published:** 2024-09-06

**Authors:** Steven P. Rowe, Rudolf A. Werner, Sangeeta Ray Banerjee

**Affiliations:** ^1^ Molecular Imaging and Therapeutics, Department of Radiology, University of North Carolina, Chapel Hill, NC, United States; ^2^ Clinic for Radiology and Nuclear Medicine, Division of Nuclear Medicine, Goethe University, Frankfurt, Germany; ^3^ The Russell H. Morgan Department of Radiology, Johns Hopkins University School of Medicine, Baltimore, MD, United States

**Keywords:** theranostics, radiotherapeutics, endoradiotherapy, PSMA, FAP

In the last decade, there has been a widespread and rapidly evolving use of the concept of “theranostics” within the field of nuclear medicine (1). Briefly, theranostics describes the *in vivo* identification of a target for therapy through the use of diagnostic imaging – most often through positron emission tomography (PET) radiotracers – and the subsequent leveraging of that target by the application of therapeutic molecules – generally β-particle- or α-particle-emitting labeled agents. In its very essence, theranostics is precision medicine in that the right patient is identified by PET uptake and the right medication is subsequently administered through a targeted theranostic (2).

The archetype of theranostics is the use of iodine-123 for imaging and iodine-131 for therapy, with such therapy having first been carried out by Saul Hertz in 1941 (3). In more recent years, we have seen that theranostic approaches for neuroendocrine tumors (4) and prostate cancer (5, 6) can be life-extending treatments. Those observations have supercharged interest in the field of nuclear medicine and emphasized the need for a robust pipeline of future theranostic agents. The clinical and pre-clinical pipelines are both brimming with potentially impactful agents that achieve optimal results by either leveraging new targets (7), utilizing radionuclides that are more energetic than currently approved agents (8), or making use of medicinal chemistry techniques to avoid toxicities from on-target or off-target binding in normal tissues (9).

Given the rapid (and accelerating) changes in the field, our special issue, titled “*Theranostics as a Driving Force in Nuclear Medicine*”, sought to engage potential authors who could provide manuscripts on a wide range of different topics across the theranostics space – from preclinical discovery through advanced clinical applications. We believe the carefully selected articles in this issue meet that objective.

For example, Chen et al. provided an excellent manuscript that described the synthesis and preclinical evaluation of a radiofluorinated PET radiotracer for imaging of the fibroblast growth factor receptor-1 (FGFR1), a target commonly found on various types of cancer. The authors found that their agent, ^18^F-FGFR1 had high uptake in RT-112 xenografts with low uptake in tissues that do not express FGFR1, thus suggesting their agent has clinical promise for imaging and therapy of FGFR1-expressing tumors.

Moving on to another preclinical topic with important implications for eventual clinical imaging, Huang et al. provided an in-depth overview of efforts to image hypoxia with PET radiotracers. The development of hypoxia in tumors portends a poor prognosis with resistance to common therapeutic methods and higher rates of recurrence and metastasis. Hypoxia imaging has the potential limitation that the low blood flow to hypoxic regions of a tumor may lead to decreased radiotracer deliver and uptake. However, the likely impending regulatory approval of an imaging agent for carbonic anhydrase IX (^89^Zr-girentuximab), a hypoxia marker in many tumors, emphasizes the need for an understanding of this group of imaging agents (10).

As a final preclinical effort, Singh et al. describe the application of prostate-specific membrane antigen (PSMA)-targeted radiotracers in models of clear cell renal cell carcinoma (ccRCC). The routine clinical use of PSMA-targeted theranostic agents in men with metastatic, castration-resistant prostate cancer, combined with the known high PSMA expression in the tumor neovasculature of ccRCC (11), makes their work a highly appealing application of theranostics. The authors report that α-particle-emitting agents provided tumor growth control and a survival advantage in murine models of ccRCC.

The scope of the manuscripts in this special issue transitions to more immediately clinically relevant work in the other submitted papers. In a manuscript from Wang and Kiess, the argument for leveraging the use of prostate-specific membrane antigen (PSMA)-targeted theranostics in non-prostate cancers is convincingly made. Numerous non-prostate cancers express PSMA – primarily in the tumor neovasculature (12). Indeed, targets such as PSMA, fibrinogen-activating protein (FAP), and FGFR1 might best be thought of as pan-cancer targets whose expression can be interrogated via PET imaging and then acted upon if appropriate levels of target are present. The authors ultimately conclude that there is theranostic promise for PSMA agents across different cancers, but that more study is needed to define appropriate patient cohorts and determine dosimetry and efficacy.

Further along the clinical spectrum, George et al. describe an optimized workflow for incorporating imaging-based dosimetry into programs utilizing lutetium-177-labeled therapeutic agents into their clinical practices. The authors’ manuscript is a great example of a practical “field guide” that includes multiple different workflows, as well as important information for anyone administering such radiopharmaceuticals. The incorporation of individualized dosimetry remains controversial, but may have an important role in personalizing doses and improving outcomes. George et al. provide important information for anyone utilizing individualized dosimetry into their practice.

Last, but certainly not least, Rahmim et al. describe the use of theranostic digital twins in order to help transition current clinical practice from a “one-size-fits-all” strategy to a paradigm in which truly precision doses are administered. There are clearly potential advantages to personalized dosimetry and to leveraging the emerging concept of digital twins. The paper by Rahmim et al. is a clear step in the right direction toward realizing the promise of such precision medicine.

We hope this collection of carefully curated manuscripts provides a flavor for the broad advances that are occurring in theranostics. Based on the successes to-date, and based on how nuclear medicine is evolving more into a theranostics-centric field, we are likely just scratching the surface of the potential of these technologies to impact patient care. Multiple coalescing trends will need to continue in order to optimize the use of theranostics for improved patient management: (1) the preclinical pipeline must continue to expand to encompass new targets and new chemical methodologies to develop molecules that bind to those targets, (2) our understanding of clinical spaces and the potential to address specific disease states with theranostic approaches must improve, and (3) our embracing of new technologies such as digital twins and artificial intelligence must be encouraged.

At the confluence of multiple revolutions in medical imaging and therapy, it is incumbent upon us to ensure that eventual reality matches the current promise.

## References

[1] Solnes LB Werner RA Jones KM Sadaghiani MS Bailey CR Lapa C. Theranostics: leveraging molecular imaging and therapy to impact patient management and secure the future of nuclear medicine. J Nucl Med. (2020) 61:311–8. doi: 10.2967/jnumed.118.220665 31924727

[2] Nikanjam M Kato S Sicklick JK Kurzrock R. At the right dose: personalised (N-of-1) dosing for precision oncology. Eur J Cancer. (2023) 194:113359. doi: 10.1016/j.ejca.2023.113359 37832506

[3] Ehrhardt JD Jr. Gulec SA. Birth of the beta-knife thyroidectomy: the radiance of saul hertz. Am Surg. (2023) 89:2145–9. doi: 10.1177/00031348211060463 35081787

[4] Strosberg J El-Haddad G Wolin E Hendifar A Yao J Chasen B. Phase 3 trial of (177)Lu-dotatate for midgut neuroendocrine tumors. N Engl J Med. (2017) 376:125–35. doi: 10.1056/NEJMoa1607427 PMC5895095 28076709

[5] Sartor O de Bono J Chi KN Fizazi K Herrmann K Rahbar K. Lutetium-177-PSMA-617 for metastatic castration-resistant prostate cancer. N Engl J Med. (2021) 385:1091–103. doi: 10.1056/NEJMoa2107322 PMC8446332 34161051

[6] Alati S Singh R Pomper MG Rowe SP Banerjee SR. Preclinical development in radiopharmaceutical therapy for prostate cancer. Semin Nucl Med. (2023) 53:663–86. doi: 10.1053/j.semnuclmed.2023.06.007 37468417

[7] Greifenstein L Gunkel A Hoehne A Osterkamp F Smerling C Landvogt C. 3BP-3940, a highly potent FAP-targeting peptide for theranostics - production, validation and first in human experience with Ga-68 and Lu-177. iScience. (2023) 26:108541. doi: 10.1016/j.isci.2023.108541 38089587 PMC10711456

[8] Kaneda-Nakashima K Shirakami Y Kadonaga Y Watabe T Ooe K Yin X. Comparison of Nuclear Medicine Therapeutics Targeting PSMA among Alpha-Emitting Nuclides. Int J Mol Sci. (2024) 25(2):933. doi: 10.3390/ijms25020933 38256007 PMC10815831

[9] Mease RC Kang CM Kumar V Banerjee SR Minn I Brummet M. An improved (211)At-labeled agent for PSMA-targeted alpha-therapy. J Nucl Med. (2022) 63:259–67. doi: 10.2967/jnumed.121.262098 PMC8805774 34088772

[10] Shuch BM Pantuck AJ Bernhard J-C Morris MA Master VA Scott AM. Results from phase 3 study of 89Zr-DFO-girentuximab for PET/CT imaging of clear cell renal cell carcinoma (ZIRCON). J Clin Oncol. (2023) 41:LBA602–LBA. doi: 10.1200/JCO.2023.41.6_suppl.LBA602

[11] Chang SS O’Keefe DS Bacich DJ Reuter VE Heston WD Gaudin PB. Prostate-specific membrane antigen is produced in tumor-associated neovasculature. Clin Cancer Res. (1999) 5:2674–81. 10537328

[12] Salas Fragomeni RA Amir T Sheikhbahaei S Harvey SC Javadi MS Solnes LB. Imaging of nonprostate cancers using PSMA-targeted radiotracers: rationale, current state of the field, and a call to arms. J Nucl Med. (2018) 59:871–7. doi: 10.2967/jnumed.117.203570 29545375

